# Influence of substrate freshness on metabolite dynamics and methane emissions from pig slurry during laboratory incubation

**DOI:** 10.1002/jeq2.70235

**Published:** 2026-08-06

**Authors:** Rui Wang, Michael Jørgen Hansen, Sasha Daniel Hafner, Frederik Rask Dalby

**Affiliations:** ^1^ Department of Biological and Chemical Engineering Aarhus University Aarhus Denmark

## Abstract

Freeze/thaw pretreatment is commonly applied to animal slurry samples in laboratory studies, but its impact on organic matter transformation and methane (CH_4_) emissions remains uncertain. In this study, slurry incubations were performed at 20°C with three treatments differing in freshness of feces, urine, and inoculum. CH_4_ production and metabolite dynamics were monitored over 30 days. Freeze/thaw of feces and urine combined with fresh inoculum yielded the highest and most stable CH_4_ production (20 g kg^−1^ volatile solids), supported by the gradual transformation of protein‐derived metabolites and no inhibition. In contrast, fully fresh material led to rapid hydrolysis and volatile fatty acids accumulation and slight inhibition of methanogenesis, whereas freezing the inoculum caused a pronounced delay likely due to impairment of methanogens. Metabolomics identified protein‐related compounds (e.g., L‐phenylalanine, 5‐aminovaleric acid, and L‐norleucine) as early substrates correlated to CH_4_ formation. In contrast, lignin/carboxyl‐rich aliphatic molecules and lipid compounds persisted as residual fractions. These results demonstrate that inoculum freshness is the decisive factor controlling freeze/thaw effects on CH_4_ production and should be prioritized when preparing substrates for laboratory incubations. When fresh materials are not practical, freeze/thaw feces and urine can substitute it when combined with fresh inoculum.

## Plain Language Summary

Methane (CH_4_) emissions from pig slurry are a major agricultural greenhouse gas source but are difficult to measure on farms, so laboratory incubations are widely used. As samples are often frozen and thawed, this study examined how freeze/thaw treatment of pig feces, urine, and slurry inoculum affects CH_4_ production and organic matter transformation at 20°C. Results showed that inoculum freshness is the primary control on CH_4_ dynamics. With fresh inoculum, freeze/thawed feces and urine produced stable CH_4_ emissions comparable to fresh materials. In contrast, freezing the inoculum delayed CH_4_ production, while fully fresh materials caused rapid organic matter degradation and short‐term inhibition linked to volatile fatty acid accumulation. Metabolite analyses indicated that protein‐derived compounds contributed to early CH_4_ formation. The study concludes that freeze/thawed feces and urine can be reliably used when combined with fresh inoculum.

**Abbreviations**
DM: dry matter
FAA: free acetic acid
TAN: total ammonia nitrogen
VFA: volatile fatty acids
VS: volatile solids

## 1 INTRODUCTION

Slurry from pig production accounts for 46% of CH_4_ emissions from manure management in Denmark, second only to cattle (52%), making it a significant source of agricultural greenhouse gas emissions (Etminan et al., 2016; Nielsen et al., 2025; Vechi et al., 2022). Accurate prediction of these emissions requires mechanistic understanding of organic matter degradation and methanogenesis. However, such mechanisms are difficult to investigate directly under farm‐scale conditions due to the complex production environment, such as variable temperatures, continuous manure accumulation, and unpredictable farm management. For this reason, laboratory batch incubations are widely used to study slurry‐derived CH_4_ formation under controlled conditions, as these conditions provide an environment for accurate investigation of microbial and biochemical processes (F. R. Dalby, Ambrose et al., 2023; van Boxmeer et al., 2026).

Logistical constraints often lead to slurry sample freezing to preserve sample content until usage and thawing of frozen material before incubation. Freeze/thaw treatment may alter substrate availability and microbial activity and thereby affect CH_4_ production. Previous studies have reported both stimulatory (Bhattad et al., 2017) and inhibitory (Abid et al., 2024; Castro et al., 2002; Meng et al., 2023) effects of freeze/thaw on methanogenesis (Gunnarsdóttir et al., 2012), depending on substrate type, inoculum, and experimental conditions (He et al., 2022; J. Li et al., 2019; Rooni et al., 2017; Wu et al., 2019). However, for pig slurry, these effects remain poorly constrained despite freezing being common in experimental workflows. In particular, most studies do not differentiate between freezing feces, urine, and inoculum, although these fractions differ substantially in chemical composition and microbial load (Trabue et al., 2016). Moreover, most freeze/thaw studies have been conducted under mesophilic conditions (35°C–37°C), representative of engineered digesters, whereas slurry in pig houses is typically stored near 20°C where microbial processes might respond differently (F. R. Dalby et al., 2021).

Beyond bulk fractions such as proteins, lipids, and fibers, pig slurry contains diverse low‐molecular‐weight compounds including amino acids, peptides, long‐chain fatty acids, and intermediate degradation products (Dadrasnia et al., 2021; L. Liu et al., 2021; Lu et al., 2019). While bulk compositional analyses can describe the general chemical makeup of the substrate, limited information on the biochemical transformations that occur during anaerobic degradation can be provided. Protein‐related fractions, in particular, are important contributors to early‐stage CH_4_ formation during batch incubation (Y. Chen et al., 2022; F. R. Dalby, Ambrose et al., 2023), but their dynamics under freeze/thaw conditions have not been addressed. Non‐targeted metabolomics provides a way to measure these compounds, offering a system‐level view of metabolic transformations (Chapleur et al., 2021; H. Chen et al., 2020; J. Liu et al., 2023; X. Wang et al., 2024).

The objectives of this study were to evaluate the effect of freeze/thaw pretreatment of feces, urine, and slurry inoculum on CH_4_ emissions and organic matter degradation at 20°C and identify key small‐molecule metabolites that might influence CH_4_ production dynamics. We hypothesized that the freshness of slurry inputs (fresh vs. freeze/thaw treatment) would alter the timing and extent of metabolite degradation, leading to distinct intermediate accumulation patterns and CH_4_ emission dynamics.

## 2 MATERIALS AND METHODS

### 2.1 Pig feces, urine and inoculum

All materials, including feces, urine, and bulk slurry, were collected separately from a full‐scale finishing pig farm in Denmark. Bulk slurry served as the inoculum, providing the active methanogenic microbial community for laboratory incubations. Feces and urine were obtained directly from finishing pigs (i.e., pigs in the final stages of production before slaughter) to ensure that the samples were not contaminated. Urine was collected during urination without floor contact, and feces were taken immediately after excretion. Feces, urine, and inoculum for the freeze/thawed treatments were collected from an initial production batch and frozen at −18°C within 1 h of collection. Three months later, fresh feces, urine, and inoculum were collected from a subsequent production batch at the same farm and used within approximately 12 h of collection. Fresh inoculum was stored at room temperature, whereas fresh feces, urine, and thawed frozen materials were stored at 4°C before incubation. All materials were homogenized before use. The 3‐month interval reflected the production cycle length at the study farm. To minimize batch effects, both batches were collected at the same growth stage under identical feeding conditions. Physicochemical characterization confirmed comparable properties between the two batches (Table 1), consistent with the stability of major physicochemical characteristics across four consecutive production batches (Figure S1).

**TABLE 1:** Key physicochemical characteristics of feces, urine, and bulk slurry.

Characteristics	Feces	Urine	Bulk slurry
Dry matter (DM) (%)	20.67 ± 0.19	1.46 ± 0.57	8.89 ± 1.79
Volatile solids (VS) (%) ^a^	86.27 ± 3.29	52.28 ± 7.46	78.60 ± 0.78
pH	6.58 ± 0.43	7.95 ± 0.46	7.14 ± 0.16
Kjeldahl N (g kg^−1^)	8.40 ± 0.78	3.20 ± 0.86	6.12 ± 1.45
NH_4_^+^‐N (g kg^−1^)	1.13 ± 0.14	0.30 ± 0.18	3.80 ± 0.92
Volatile fatty acids (g kg^−1^)	6.63 ± 0.90	ND	13.29 ± 1.03
Cellulose (g kg^−1^)	29.57 ± 1.15	ND	13.85 ± 4.64
Hemicellulose (g kg^−1^)	45.86 ± 0.51	ND	20.48 ± 2.92
Lignin (g kg^−1^)	12.99 ± 0.68	ND	4.57 ± 1.21

*Note*: The dry matter (DM) and volatile solids (VS) content of the materials were measured before and after freezing (*n* = 3 per condition), and the standard errors reflect the differences between the two conditions (*n* = 6 in total).

^a^ Percentage of dry matter.

Abbreviation: ND, not determined.

### 2.2 Experiment design

#### 2.2.1 Substrate freshness experiment

To evaluate the effects of substrate freshness on CH_4_ production, a batch incubation experiment was conducted using three treatments differing in the freshness of feces, urine, and inoculum. The treatments included: (1) fresh feces and urine with fresh inoculum (FF); (2) thawed feces and urine with fresh inoculum (TF); and (3) thawed feces and urine with thawed inoculum (TT). In Danish pig houses with vacuum flushing, slurry is typically discharged weekly (Regeringen, 2024). Thirty days of incubation was conducted to capture both short‐ and long‐ retention scenarios, enabling results to be interpreted in the context of different manure management in pig houses (F. R. Dalby, Hansen et al., 2023).

All treatments were performed in triplicate at room temperature (20°C). Sampling was carried out at the start and after 2, 14, and 30 days. At Days 2, 14, and 30, three bottles per treatment were sacrificed for physicochemical and metabolomic analyses. Only the bottles incubated for 30 days were used for CH_4_ quantification, as these remained undisturbed until the end of incubation. A urine‐to‐feces ratio of 3:1 (as‐sampled mass basis) was adopted based on F. R. Dalby et al. (2020), while the inoculum proportion (25% as‐sampled mass basis) was selected based on a preliminary experiment (Figure S2), in which this amount produced the highest CH_4_ yield among the tested levels. Fresh and thawed inoculum controls were included, and their CH_4_ production was subtracted to obtain net feces‐ and urine‐based CH_4_ yields.

#### 2.2.2 Free acetic acid inhibition experiment

To assess the inhibitory effect of acetic acid on methanogenesis, acetic acid was added to slurry samples collected from the same location as in the substrate freshness experiment. Acetic acid was selected because it is the predominant volatile fatty acids (VFA) in pig slurry and a direct substrate for acetoclastic methanogens. Final acetic acid concentrations were 0, 1, 2, 3, 4, 5, 6, 8, and 10 g L^−1^, with each concentration prepared in duplicate. All incubations were carried out at 20°C under anaerobic conditions. A 4‐day incubation captured the short‐term methanogenic response to acetic acid.

The free acetic acid (FAA) concentration in each treatment was calculated according to Equation (1), based on the externally added acetic acid concentration and the pH measured immediately after preparation:

(1) $$\mathrm{FAA}\;=\frac{C_{\mathrm{added}}\times C_{H^{+}}}{K_{a}+C_{H^{+}}}\;$$

where FAA is the free acetic acid concentration (g L^−1^), $C_{\mathrm{added}}$ is the acetic acid addition concentration (g L^−1^), $C_{H^{+}}$ is the proton concentration (g L^−1^) estimated as the antilog of negative measured pH, and $K_{a}$ is the dissociation constant of acetic acid.

### 2.3 Substrate freshness experiment setup

**FIGURE 1:**
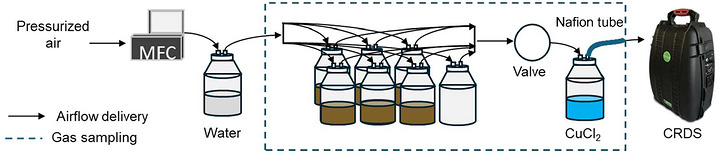
Experimental setup.

To simulate real farm ventilation conditions, a continuous‐flow system was used to monitor CH_4_ emissions from 39 individual bottles under controlled conditions. Glass bottles (DURAN BlueCap flasker, DWK Life Sciences) with a 0.5 L total volume were fitted with ports (GL14, DWK Life Sciences) for air inlet and gas outlet. The experimental setup comprised three main components: (i) an airflow delivery system, (ii) a gas sampling and distribution system, and (iii) analytical instrumentation (Figure 1).

Pressurized airflow was controlled using mass flow controllers (Bronkhorst EL‐FLOW) and humidified by passing through a bottle containing demineralized water to minimize slurry evaporation. The humidified air was distributed to each reactor headspace through 1/8‐inch PTFE tubing, delivering a stable airflow of 50  ±  2 mL min^−1^ (20°C, 1.0 atm) per reactor, as confirmed by a flowmeter (TSI Series 4100). Of the 39 reactors, 15 were used for 30‐day CH_4_ quantification. These included three replicates each of FF, TF, TT, fresh inoculum only, and thawed inoculum only. One additional background reactor was also included, giving a total of 16 monitored reactors connected to the gas analysis system. The remaining reactors were used for physicochemical and metabolomic sampling on Days 2 and 14 and were connected only to the airflow system, and the outlet gas directed entirely to exhaust.

Headspace gas from the 16 monitored reactors was sampled continuously using a Picarro A0311 16‐port distribution manifold equipped with a rotary valve system (Picarro Inc.). Each reactor was measured for 25 min, resulting in a complete measurement cycle of approximately 6.7 h. During measurement, the outlet gas stream was split using a needle valve. Gas was supplied to the CRDS analyzer at 30 mL min^−1^, while the remaining 20 mL min^−1^ was exhausted. The first 15 min of each sampling period were used for flushing and signal stabilization to minimize memory effects between reactors. Only the final 10 min, during which the signal was stable, were averaged for CH_4_ analysis. The outlet gas stream was pretreated by passing through a CuCl_2_ scrubber and a Nafion dryer (MD‐110‐48P‐4, Perma Pure LLC) to remove hydrogen sulfide, water vapor, and other gases that can cause spectral interferences (García et al., 2024).

CH_4_ concentrations were then measured using cavity ring‐down spectroscopy (CRDS, Picarro G4301). More than 95% of measured concentrations fell below 100 ppm (Figure S3A), well within 0–800 ppm range of instrument (Picarro, 2021). Therefore, all measurements were well within the reliable operating range of the instrument. In addition, prior to the experiment, the mass flow controllers, mass flow meter, and the Picarro G4301 were calibrated using a reference flowmeter and certified CH_4_ standard gases, respectively. Calibration results confirm good linearity and accuracy (*R* ^2^ = 0.996–0.999) within the concentration range encountered in this study and are provided in the Supporting material (Figure S3B–D).

### 2.4 Analytical methods

#### 2.4.1 Physicochemical analyses

At each sampling event, slurry samples were divided into two portions for physicochemical and chemical analyses. The first portion was used to determine dry matter (DM), volatile solids (VS), and fiber components. DM was determined by drying samples at 105°C for 24 h. For fiber analysis, samples were dried at 60°C for 96 h to minimize structural alteration, and a subsample was milled using a ball mill (MM 400, Retsch GmbH) at 5000 rpm for 30 s. Fiber fractions, including neutral detergent fiber (NDF), acid detergent fiber (ADF), and acid detergent lignin (ADL), were analyzed using the Van Soest method (Van Soest et al., 1991). This method separates plant fiber components based on detergent solubility. VS was determined by combusting dried samples at 550°C until constant weight.

The second portion was used for pH measurement and chemical analyses, including total nitrogen (TN), total Kjeldahl nitrogen (TKN), total ammonia nitrogen (TAN), and VFA. Total nitrogen was determined using different analytical approaches depending on sample composition: TN was measured directly for treatments containing inoculum, whereas TKN was determined for feces–urine mixtures without inoculum. TAN and VFA were quantified using standard photometric and gas chromatographic methods, respectively. Detailed analytical procedures are provided in Supporting Information S1.

#### 2.4.2 Non‐targeted metabolic profiling

Approximately 15 g of slurry was rapidly frozen in liquid nitrogen and subsequently lyophilized for 72 h. For extraction, 250 mg of freeze‐dried material was mixed with 3 mL of methanol:water (4:1, v/v), vortexed, centrifuged twice, and filtered through a 0.22‐µm membrane. Extracts were diluted fivefold with deionized water prior to LC–MS analysis to reduce matrix effects (Stahnke et al., 2012). Metabolomic analysis was performed using a Vanquish UHPLC system coupled to an Orbitrap Exploris 120 mass spectrometer (Thermo Fisher Scientific). Detailed chromatographic and mass spectrometry parameters are provided in Supporting Information S2.

### 2.5 Data processing and statistics

All analyses used R (version 4.3.2), Microsoft Excel, Compound Discoverer 3.3, and MetaboAnalyst 6.0.CH_4_ data and slurry physicochemical parameters were processed using R and Excel. CH_4_ concentration data underwent background correction, emission rate calculation, and time‐based integration in R to obtain daily and cumulative CH_4_ emissions. Slurry properties (e.g., pH, TAN, and VFA) were sorted and summarized in Excel. Statistical significance was defined as *p* < 0.05.

To minimize the potential false positives arising from multiple daily comparisons, cumulative CH_4_ emissions were analyzed only at representative incubation days (Days 7 and 30) using one‐way analysis of variance (ANOVA), with treatment included as a fixed factor. Post hoc pairwise comparisons among treatments were conducted using Tukey's HSD test (*α* = 0.05).

Metabolomic data were processed in Compound Discoverer 3.3 for peak detection, alignment, deconvolution, and metabolite annotation. The resulting compound table was used for downstream multivariate and pathway‐level analyses in MetaboAnalyst 6.0. Sparse partial least squares discriminant analysis (sPLS‐DA) was applied to identify treatment‐specific metabolic profiles. Peak areas were log‐transformed and auto‐scaled prior to analysis. Metabolites were classified into major chemical groups based on elemental ratios using a rule‐based system implemented in R (Table S1). For correlation analysis, metabolomic data were obtained from pooled samples collected at Days 0, 2, 14, and 30, while TAN, VFA, and daily CH_4_ production rates were derived from replicate incubations.

## 3 RESULTS

### 3.1 Methane emission

**FIGURE 2:**
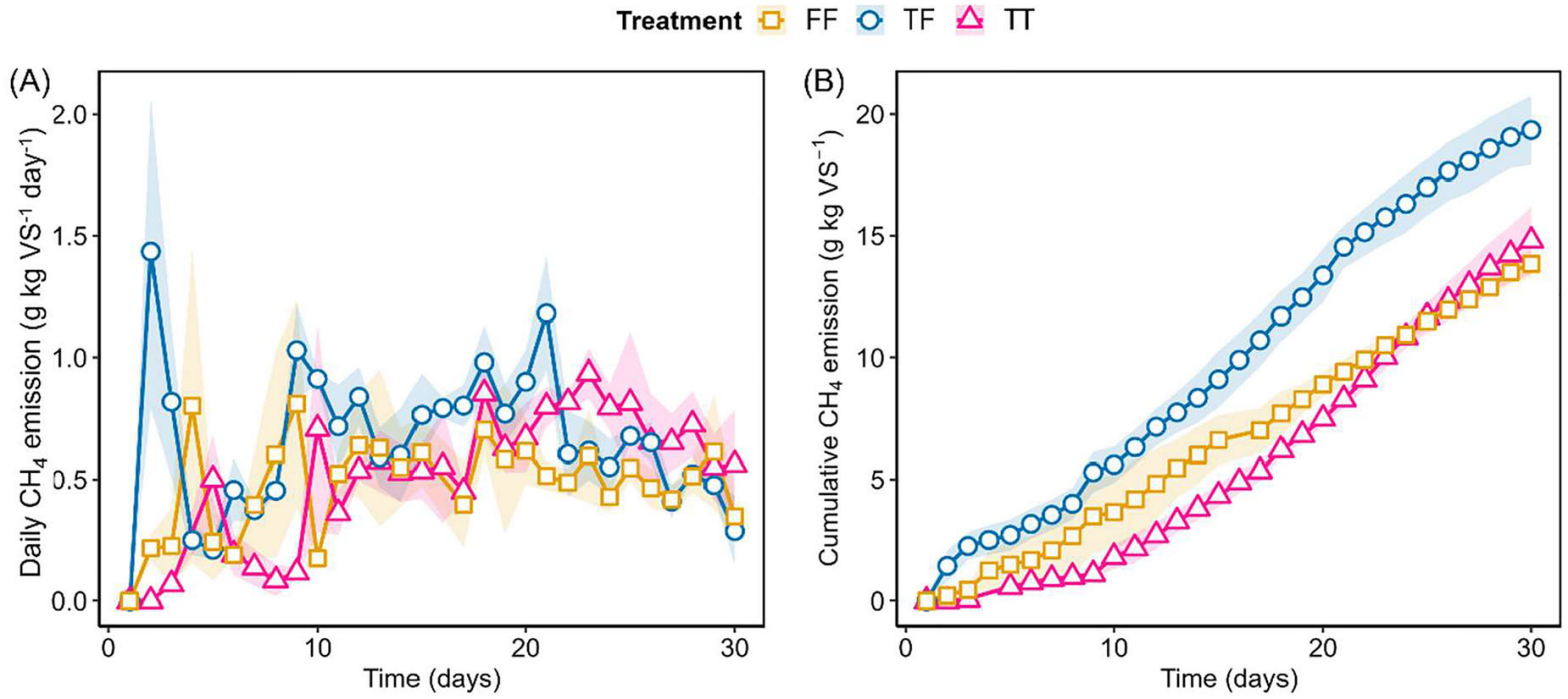
Daily (A) CH_4_ and cumulative (B) CH_4_ emissions under treatments with varying material freshness. FF, fresh feces, urine, and inoculum; TF, freeze/thaw feces, urine, and fresh inoculum; TT, freeze/thaw feces, urine, and inoculum. Data are presented as mean ± SE (*n* = 3).

Freeze/thaw influenced the onset, intensity, and duration of CH_4_ emission during the 30‐day incubation (Figure 2). Across all treatments, CH_4_ production rate generally exhibited an initial increase followed by a stabilization or decrease phase, and the timing and magnitude varied with substrate freshness. Treatments with fresh inoculum (FF and TF) exhibited higher early‐stage CH_4_ production rates than the treatment in which both substrate and inoculum were freeze/thawed (TT). In TF, CH_4_ production peaked initially, declined, and then increased again during the mid‐incubation phase (Days 7–15), followed by a gradual decrease. A similar pattern was observed in FF, where CH_4_ production increased with some fluctuations until Day 17 and did not differ significantly from TF during this period (Figure 2A). In contrast, CH_4_ production in TT remained low during the first 22 days and increased only during the late incubation phase. As a result, TF achieved the highest cumulative CH_4_ production over 30 days (19.72 g kg^−1^ VS_added_), which was significantly higher than that of the FF and TT treatments at the end of the incubation (*p* < 0.05). However, no significant difference was observed in cumulative emissions between FF and TT by the end of incubation (*p* > 0.05).

### 3.2 Slurry properties

**FIGURE 3:**
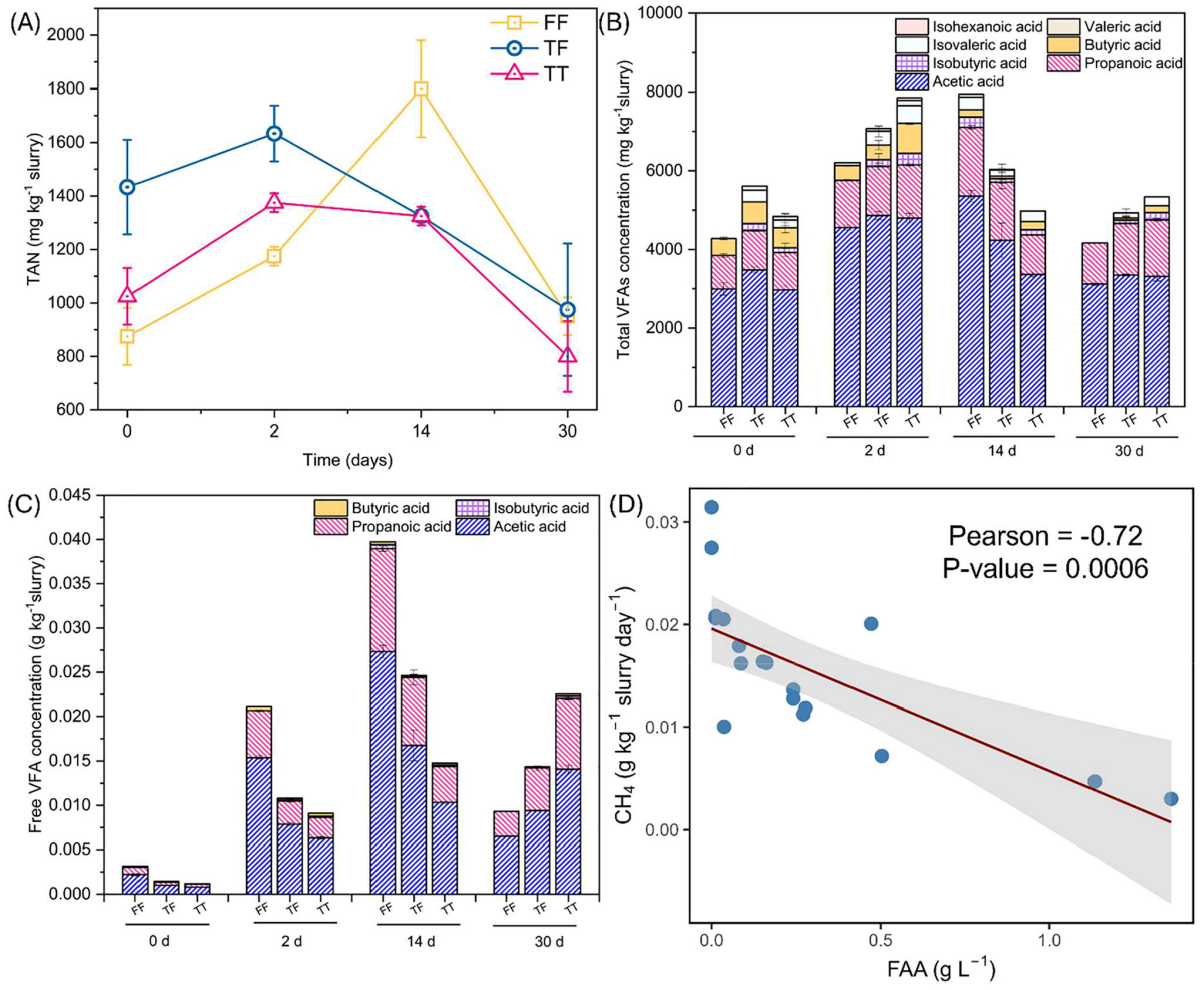
Dynamic changes in TAN (A), total VFA (B), and free VFA (C) under different material freshness treatments, and the correlation between free acetic acid and CH_4_ production from a separate experiment, but with manure from the same farm (D). The error bands surrounding the regression lines represent the 95% confidence interval of the correlation (D). All treatments and their operational parameters are detailed in captions of Figure 2. VFA, volatile fatty acids.

To investigate potential inhibitory or stimulatory effects on methanogenesis, the dynamics of TAN, total VFA, and free VFA were analyzed across treatments (Figure 3). All three parameters generally increased initially and declined thereafter, with FF showing the most prolonged accumulation.

While TAN levels increased during the early stage (Day 0–2) in all treatments, the timing and magnitude of peak accumulation varied considerably (Figure 3A). TF exhibited the fastest accumulation, while FF showed a delayed yet more pronounced increase, resulting in significantly higher TAN levels than TF and TT on Day 14 (*p* < 0.05). By Day 30, TAN concentrations had declined and converged across treatments without significant differences.

A comparable trend was observed for total VFA (Figure 3B), although the temporal dynamics differed among treatments. TT reached its peak earlier, whereas FF exhibited a delayed accumulation and maintained significantly higher concentrations on Day 14 compared to the other treatments (*p* < 0.05). TF showed an intermediate trend, with a quicker rise and earlier decline. At the end of the incubation, TT retained relatively higher VFA levels.

Free VFA, representing the main inhibitory fraction, followed a comparable increase–decrease pattern across treatments (Figure 3C). FF reached the highest peak on Day 14, significantly exceeding TF and TT, whereas by Day 30 free VFA concentrations declined in all treatments, with TT retaining the highest residual levels. A significant negative correlation was observed between FAA concentration and cumulative CH_4_ production (*r* = −0.76, *p *= 0.0011; Figure 3D).

### 3.3 Overall metabolite composition and differences

**FIGURE 4:**
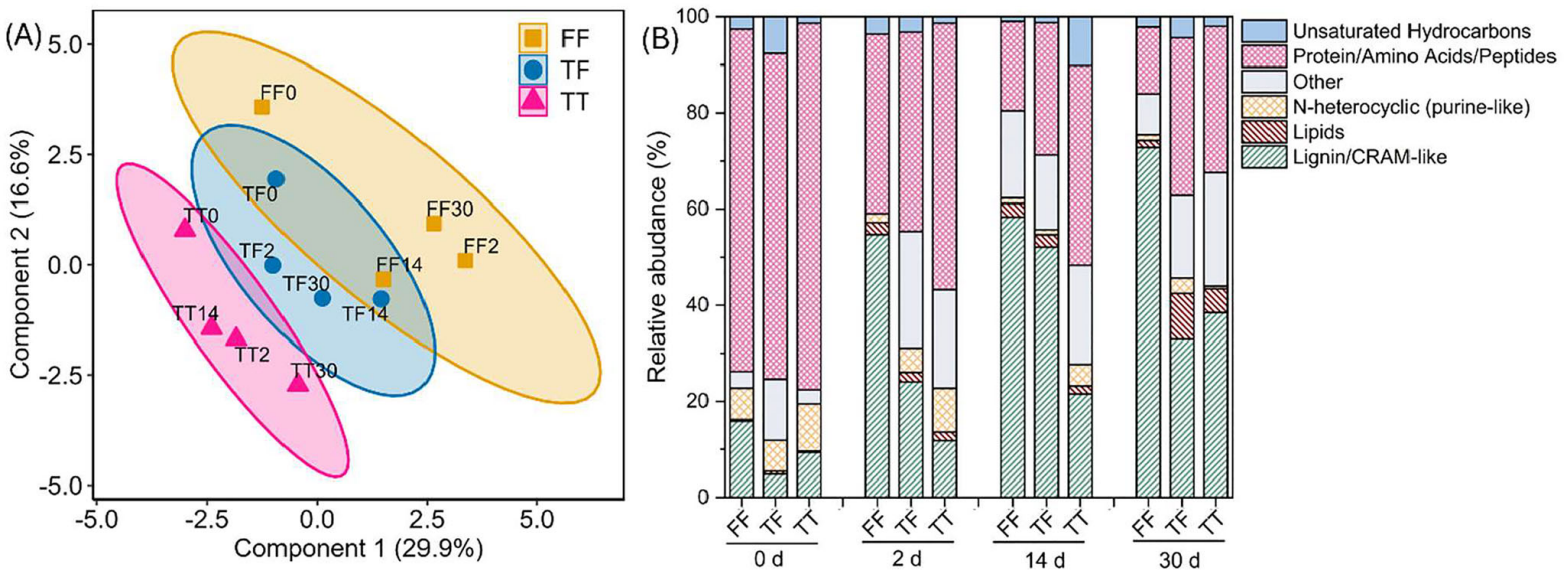
Sparse partial least squares discriminant analysis (A) and compound composition (B) across all treatments under different material freshness treatments. Their operational parameters are detailed in the captions of Figure 2.

To link observed TAN and VFA dynamics with underlying substrate transformation, further analysis was conducted to investigate the changes in overall metabolite composition during incubation. A total of 338 metabolites were detected in all slurry samples. The sPLS‐DA score plot (Figure 4A) revealed partial separation among the FF, TF, and TT treatments, indicating that freeze/thaw influenced the overall metabolic composition. FF and TT were located at opposite ends of the first component, while TF appeared in an intermediate position, indicating that freeze/thaw treatment altered overall metabolic composition.

All 338 metabolites were classified into chemical groups by elemental ratios (C, H, O, N, and P) using a rule‐based system (Table S1), independent of treatment or time point. Functional classification (Figure 4B) showed that protein/amino acids/peptides were the dominant group (35.9%), followed by lignin/CRAM‐like compounds (27.4%), N‐heterocyclic (purine‐like) (3.53%), unsaturated hydrocarbons (2.80%), lipids (1.98%), and others. Over time, proteins and peptides generally declined in relative abundance across all treatments, while lignin/CRAM‐like and lipid compounds increased progressively. By Day 30, protein‐derived compounds had declined most markedly in FF.

### 3.4 Temporal metabolite dynamics

**FIGURE 5:**
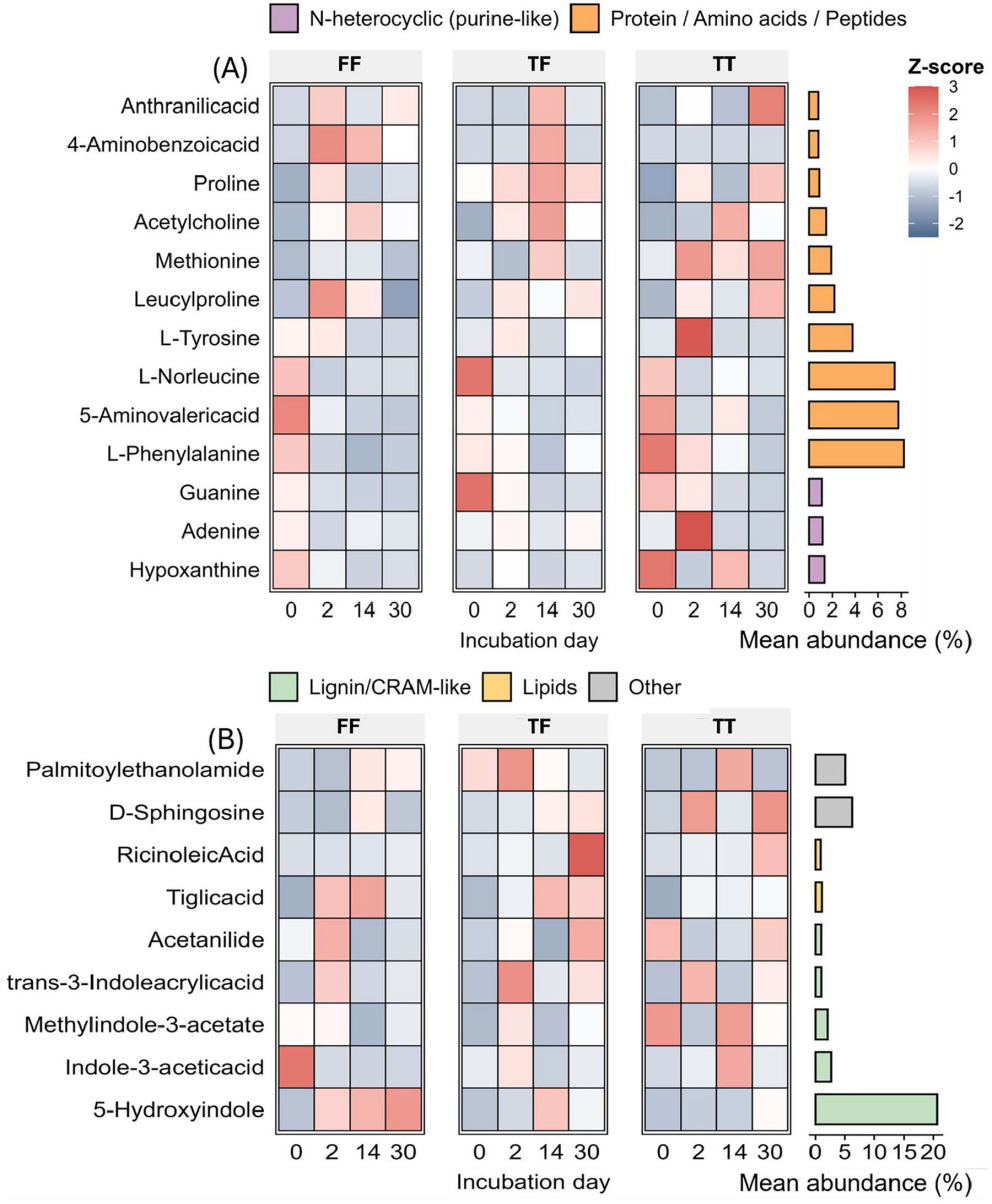
Protein‐related and purine metabolites succession (A) and lignin/CRAM‐like compounds, lipids, and other metabolites succession (B). Their operational parameters are detailed in the captions of Figure 2. Z‐scores represent standardized metabolite abundances calculated relative to each compound's mean and standard deviation; red indicates higher‐than‐average abundance, blue indicates lower‐than‐average abundance, and color intensity reflects the degree of deviation.

The temporal dynamics of 22 representative metabolites, accounting for 72%–94% of the total metabolite peak area, were examined across all treatments and grouped into five functional categories dominated by protein‐related and lignin/CRAM‐like compounds (Figure 5).

At the start of incubation, the freeze/thawed substrate with fresh inoculum (TF) showed higher abundances of several low‐molecular‐weight metabolites than the fully fresh treatment (FF), particularly protein‐related compounds (e.g., anthranilic acid and 4‐aminobenzoic acid) and the purine compound guanine. Compared with TF, the fully freeze/thawed treatment (TT) exhibited even higher initial abundances of representative metabolites such as L‐phenylalanine, 5‐aminovaleric acid, guanine, and hypoxanthine. Despite these initial differences, metabolites followed two contrasting temporal patterns during incubation. One group, consisting mainly of protein‐related and purine metabolites, declined over time (Figure 5A), whereas a second group, including lignin/CRAM‐like compounds, lipids, and other metabolites, generally accumulated during incubation (Figure 5B).

Among protein‐related compounds, most metabolites decreased throughout incubation, although degradation rates differed among treatments. L‐phenylalanine and 5‐aminovaleric acid decreased most rapidly in TT and FF during the first 2 days, whereas their decline was slower in TF. Conversely, L‐norleucine decreased fastest in TF. Several other amino acid derivatives (e.g., anthranilic acid, 4‐aminobenzoic acid, proline, and acetylcholine) showed initial accumulation followed by depletion, with treatment‐specific timing. In FF, most amino acid derivatives reached peak concentrations by Day 2. In TF, metabolite accumulation continued until Day 14 before declining, whereas in TT, metabolite concentrations fluctuated and remained relatively high until Day 30. Purine‐like compounds showed a similar but slightly shifted pattern, with the highest initial abundances in TT and the most pronounced early‐stage degradation in FF.

Lignin/CRAM‐like compounds were dominated by 5‐hydroxyindole, which increased consistently throughout incubation, particularly in TF and FF (Figure 5B). Other lignin/CRAM‐like metabolites showed more complex dynamics. In TT, methylindole‐3‐acetate and indole‐3‐acetic acid started at relatively high levels, declined rapidly, showed transient accumulation around Day 14, and then decreased again. In FF, these metabolites declined more steadily, whereas in TF they exhibited fluctuating patterns before stabilizing. Several compounds, including acetanilide and trans‐3‐indoleacrylic acid, accumulated over time across all treatments. Similar increasing tendencies were observed for lipid‐derived compounds such as ricinoleic acid and tiglic acid, although their trajectories differed among treatments.

### 3.5 Correlation between protein‐related metabolites, intermediates, and CH_4_ production

**FIGURE 6:**
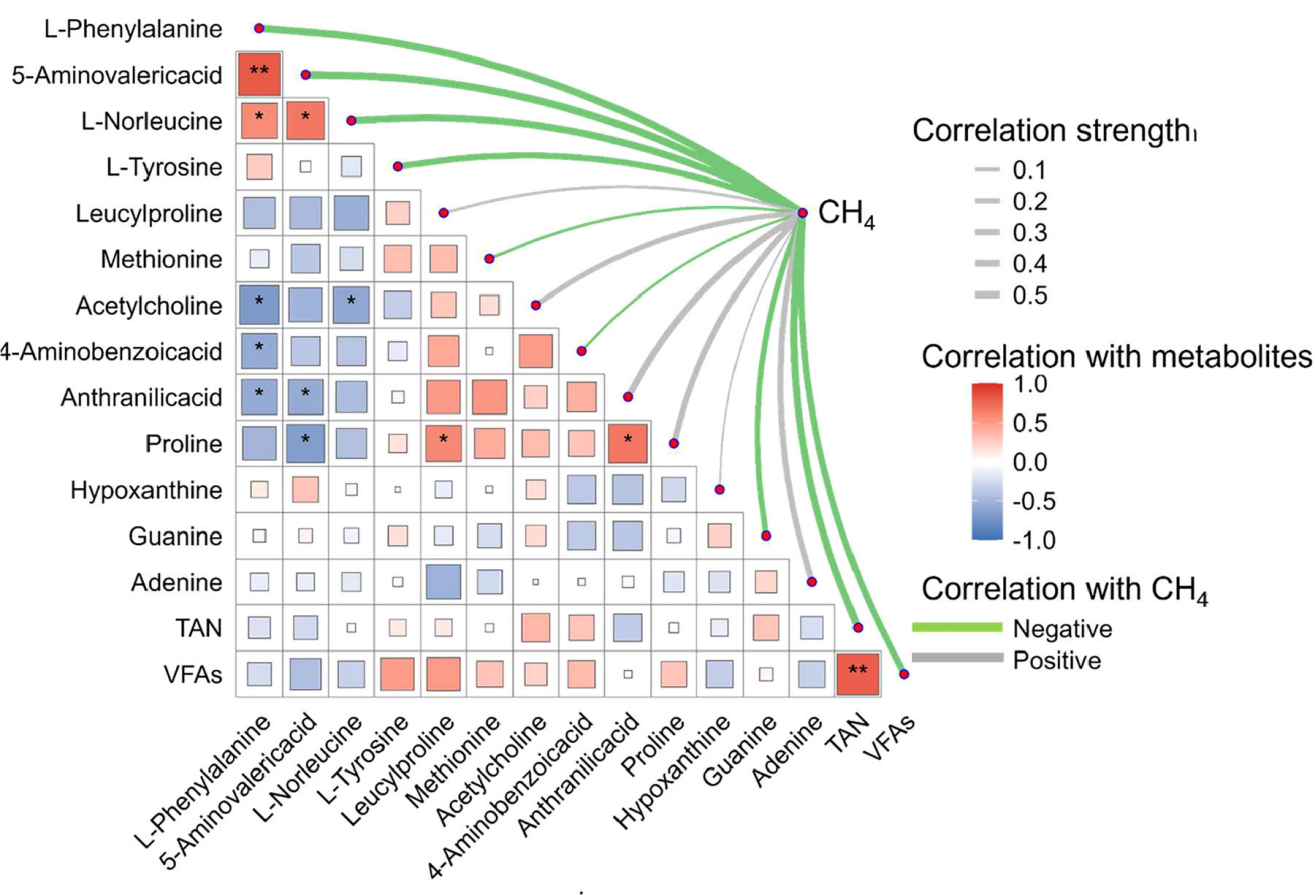
Correlations between representative metabolites and daily CH_4_ production rates. Heatmap cells show pairwise correlation coefficients among metabolites, TAN, and VFA, with color indicating correlation direction and strength. Curved lines indicate correlations between individual metabolites and CH_4_, with line thickness representing correlation strength and color indicating positive (gray) or negative (green) correlations. Metabolomic data were obtained from pooled samples, in which material from three replicate bottles was combined at each sampling time point (d 0, 2, 14, and 30). TAN, VFA, and daily CH_4_ production rates were measured in replicate bottles, and mean values corresponding to the same sampling day were used for correlation analysis. Asterisks denote statistically significant correlations (*p < 0.05, **p < 0.01). TAN, total ammonia nitrogen; VFA, volatile fatty acids.

Since protein‐related and purine metabolic pathways were the most frequently detected, Pearson correlation analysis was further performed to explore potential organic decomposition drivers of CH_4_ production. Correlation heatmaps were constructed between protein‐related and purine metabolites, TAN and VFA, and CH_4_ production rate (Figure 6). High‐abundance protein‐related metabolites, including L‐norleucine, L‐phenylalanine, 5‐aminovaleric acid, and L‐tyrosine, exhibited negative correlations (*r *= −0.52 to −0.33) with CH_4_, and most of them also showed positive correlations with VFA and TAN except L‐Tyrosine. In contrast, several low‐abundance amino acid derivatives (e.g., anthranilic acid and 4‐aminobenzoic acid) showed positive correlations with CH_4_ but weak or negative associations with VFA and TAN. Among purine‐related metabolites, adenine and hypoxanthine were positively correlated with both CH_4_ and TAN, while guanine displayed a negative correlation with CH_4_ and a weaker correlation with TAN.

## 4 DISCUSSION

### 4.1 Comparative CH_4_ production patterns across treatments

The observed freeze/thaw effects on slurry methanogenesis appear to be influenced by inoculum freshness. The stable and sustained CH_4_ production observed in treatment with fully freeze/thaw substrates and fresh inoculum (TF) likely reflects preserved microbial activity combined with a gradual release of labile substrates from freeze/thaw material, thereby maintaining balance between acidogenesis and methanogenesis and preventing inhibitory accumulation. In contrast, methanogenesis in treatment with fully fresh substrates and inoculum (FF) exhibited rapid initial CH_4_ formation followed by a moderate slowdown after approximately two weeks. This result may be associated with accelerated hydrolysis of fresh feces and urine and a transient accumulation of VFA. The delayed onset of CH_4_ production in treatment with fully freeze/thaw substrates and inoculum (TT) is consistent with previous observations of freeze/thaw‐induced impairment of anaerobic microbial activity. A separate experiment supported this interpretation. Fresh and freeze/thawed inocula differed significantly on Day 7 (*p* < 0.001) but not on Day 30 (*p* = 0.554; Figure S4), likely indicating only a transient suppression of methanogenic activity. Castro et al. (2002) also reported that freezing at −20°C for 2–5 months reduced the specific methanogenic activity of anaerobic sludge by 29%–85%. This impairment is likely related to selective damage to hydrogenotrophic methanogens, which have been shown to be particularly sensitive to cold storage compared to acetoclastic methanogens (Hagen et al., 2015). Hydrogenotrophic methanogenesis has been reported as the dominant pathway in pig slurry at ambient temperatures (F. R. Dalby, Ambrose et al., 2023), and the selective impairment of this functional group likely underlies the pronounced lag phase observed in the frozen inoculum. Although this interpretation is supported by previous studies and the observed CH_4_ production dynamics, direct evidence was not available in this study because no microbial community analyses were conducted.

Although TF achieved significantly higher cumulative CH_4_ production after 30 days than the other treatments, CH_4_ production from TF and FF did not differ during the first 7 days (Figure 2). This suggests that TF can represent early‐stage slurry degradation under farm conditions. Due to mandatory weekly discharge at Danish fattening pig farm, comparisons beyond the first week might have limited practical relevance. However, TT appears less suitable for simulating in situ CH_4_ dynamics, as reflected by its slower initial degradation despite comparable cumulative CH_4_ emissions to FF at incubation end. The similar cumulative CH_4_ production of FF and TT likely resulted from contrasting trajectories, with rapid initial methanogenesis followed by transient inhibition in FF, and delayed methanogenic recovery in TT.

### 4.2 Mechanism of organic degradation

The distinct CH_4_ production patterns observed across treatments were closely linked to differences in hydrolysis intensity, intermediate accumulation, and methanogenic recovery, which were primarily controlled by substrate and inoculum freshness.

In the TF treatment, metabolite behavior indicates that the fresh inoculum maintained microbial activity while the freeze/thawed substrate enhanced solubilization of organics without causing substrate overloading. At the start of incubation, the TF treatment showed a higher abundance of amino acids (such as L‐norleucine) and the purine‐related compound guanine. This likely reflected the physical release of intracellular and protein‐bound metabolites during freezing and thawing. Also, the gradual and sequential depletion of protein‐related metabolites during incubation indicates a temporally distributed substrate supply, which helped maintain a balance between acidogenesis and methanogenesis. The persistence of lignin/CRAM‐like and lipid‐derived compounds (e.g., 5‐hydroxyindole and ricinoleic acid) is consistent with their low degradability at 20°C. These compounds mainly represent residual pools with limited contribution to CH_4_ formation, in agreement with previous observations for aromatic (Sengupta et al., 2001) and lipid compounds that hydrolyze slowly at 20°C (Béline et al., 1998).

In contrast, the instability observed in FF was likely caused by rapid substrate release from fresh feces and urine. Protein‐derived metabolites were almost depleted during the first 2 days, releasing soluble organics faster than methanogens could utilize them. This is supported by F. R. Dalby, Ambrose et al. (2023), who reported a significant decline (10%) in crude protein in pig slurry within the first 2 days of incubation at 20°C, concurrent with rapid VFA accumulation. The resulting excess supply of intermediates was accompanied by VFA accumulation, which is consistent with previous findings that excessive acidogenesis can inhibit methanogenesis (W. Zhang et al., 2018, 2019) by lowering pH and disturbing proton balance (Kashket, 1985; Puchajda & Oleszkiewicz, 2006; Roe et al., 1998).

The delayed CH_4_ production observed in TT highlights the sensitivity of the inoculum to freeze/thaw treatment. Although freezing likely released intracellular substrates from lysed microbial cells (Montusiewicz et al., 2010; She et al., 2020), the rapid depletion of soluble metabolites during the first day suggests that hydrolytic and acidogenic bacteria recovered faster than methanogens (Hagen et al., 2015). The resulting accumulation of VFA indicates that methanogenic activity remained impaired for an extended period, consistent with earlier reports of freeze/thaw‐induced damage to anaerobic consortia (Castro et al., 2002; Hagen et al., 2015; Koch et al., 2019).

### 4.3 Potential indicator significance of metabolites

Correlation analysis was performed to identify potential indicators of methanogenic activity. Across treatments, the main protein‐related metabolites detected by non‐targeted metabolomics were negatively correlated with CH_4_ production, indicating that their depletion might be generally associated with methanogenic progression. In particular, L‐phenylalanine, L‐tyrosine, 5‐aminovaleric acid (obtained from L‐lysine degradation) (Cardona et al., 2020), and L‐norleucine showed negative correlations with CH_4_ production. Amino acids such as phenylalanine, lysine, and tyrosine were reported to enter the acetyl‐CoA biosynthesis pathway (Niu et al., 2025; Teufel et al., 2010; S. Wang et al., 2022), producing pyruvate and acetyl‐CoA that act as precursors for VFA (Schröder et al., 1994) and as direct methanogenic substrates. This metabolic role is consistent with their observed negative correlations with CH_4_, where rapid depletion may reflect periods of enhanced methanogenic activity, whereas their persistence indicates delayed or inhibited conversion. For example, elevated L‐tyrosine concentration in TT on Day 2 may reflect reduced microbial activity and slower organic matter conversion to CH_4_.

Several amino acid derivatives, particularly anthranilic acid, proline, and acetylcholine, exhibited positive correlations with CH_4_ and showed treatment‐specific accumulation and depletion patterns. Anthranilic acid, an intermediate in tryptophan degradation, can be further transformed into small organic acids under anaerobic conditions (Shaw et al., 2023; Xiao et al., 2024). Proline, which is involved in the arginine and proline metabolism pathway, can enhance VFA production by serving as an electron donor in the reductive degradation of aromatic intermediates such as indole (X. Li et al., 2018). Acetylcholine can be hydrolyzed to choline and acetate, serving as a source of acetate under methanogenic conditions (Watson et al., 2012). The transient accumulation of these metabolites may therefore reflect coordinated interactions between amino acid turnover, redox regulation, and methanogenic activity. Similarly, recalcitrant compounds such as ricinoleic and tiglic acids and lignin/CRAM‐like molecules including 5‐hydroxyindole also showed apparent positive correlations with CH_4_. These relationships may indicate depletion of labile substrates and a shift toward late‐stage slurry degradation rather than direct participation in methanogenesis.

Overall, these correlation patterns indicate consistent associations between amino‐acid metabolism, intermediate dynamics, and methanogenic activity. Although correlation does not imply causality, the repeated co‐variation of key metabolites with CH_4_ production suggests that they may serve as potential indicators for monitoring manure degradation efficiency. However, as relative abundances based on peak areas do not represent absolute concentrations, targeted quantification of key metabolites will be required to assess their quantitative contributions to CH_4_ formation.

### 4.4 Representativeness of batch incubations across laboratory and full‐scale systems

Compared to the reported results, the mean cumulative CH_4_ emissions over 30 days across the three treatments (FF, TF, and TT) observed in this study (averaging 0.54 g kg^−1^ VS day^−1^) were intermediate between uninoculated manure and well‐adapted slurry inocula. For example, higher emissions have been reported for slurry with longer in‐barn retention times (1.30–1.97 g kg^−1^ VS day^−1^) in Hilgert et al. (2022) and Petersen et al. (2016) whereas lower emissions around 0.33 g kg^−1^ VS day^−1^ were obtained from freeze/thawed feces and urine incubated without inoculum at 20°C (F. R. Dalby, Ambrose et al., 2023). These differences are consistent with the role of inoculum availability and methanogenic adaptation in controlling CH_4_ production. In addition, longer slurry retention in storage pits has been shown to enhance methanogenic activity and improve VFA conversion efficiency (Lendormi et al., 2022; Steinmetz et al., 2016).

Considering the uncertainty in VS content from farm, CH_4_ emissions were compared to farm‐scale values on a slurry mass basis (w/w). During laboratory incubation, CH_4_ emissions averaged 0.026 ± 0.0046 g kg^−1^ slurry·day^−1^, which is slightly lower than most values reported for corresponding finishing pig houses with weekly flushing (average: 0.049 g kg^−^^1^ slurry day^−1^), and other reports range of 0.01–0.17 g kg^−1^ slurry day^−1^ (F. R. Dalby, Hansen et al., 2023; Ni et al., 2008; Petersen et al., 2024). This difference likely reflects microbial and substrate‐related contrasts between laboratory and on‐farm systems. Oxygen exposure during slurry handling may reduce methanogenic activity (Yu et al., 2020), and closed‐batch incubations involve no continuous substrate input, leading to gradual substrate depletion and declining microbial activity. In contrast, farm‐scale systems maintain a continuously renewed microbial community and receive additional soluble organic matter from feed spillage, which can substantially increase slurry pit CH_4_ emissions (F. R. Dalby et al., 2024; X. Wang et al., 2024; Lashkari et al., 2025).

Overall, the slightly lower CH_4_ emissions observed in batch incubations likely reflect their limited ability to reproduce key farm‐scale processes, including continuous manure input, microbial adaptation, inoculum dynamics, and oxygen exposure during slurry handling. As these factors can substantially influence CH_4_ emissions in commercial manure storage systems (F. R. Dalby, Ambrose et al., 2023, F. R. Dalby et al., 2024), batch incubation results should be interpreted cautiously when extrapolating to farm‐scale conditions. Recent continuous laboratory systems incorporate continuous substrate input and microbial adaptation, allowing dynamic processes to be represented under controlled conditions (Rawat et al., 2026). Future studies could adopt such systems to better reflect on‐farm slurry system.

## 5 CONCLUSION

This study examined the effect of substrate freshness on organic metabolite degradation and CH_4_ production dynamics in pig slurry. When fresh feces and urine are unavailable, thawed feces and urine with fresh inoculum should be used, because fresh inoculum better reproduces in situ slurry dynamics than thawed inoculum. Protein‐derived compounds such as L‐phenylalanine, 5‐aminovaleric acid, and L‐norleucine are associated with key early methanogenic substrates that promote CH_4_ formation. CH_4_ emissions from the applied batch incubations were slightly lower than those observed under farm conditions. From a methodological perspective, incorporating continuous or semi‐continuous manure input could better simulate the dynamic environment of on‐farm slurry systems. Future research should integrate targeted metabolomics with microbial community analysis to enable absolute quantification of key degradable protein‐derived metabolites and to validate these potential biochemical indicators under representative field conditions.

## AUTHOR CONTRIBUTIONS

**Rui Wang**: Conceptualization; data curation; formal analysis; investigation; methodology; software; validation; visualization; writing—original draft; writing—review and editing. **Michael Jørgen Hansen**: Investigation; project administration; supervision; writing—review and editing. **Sasha Daniel Hafner**: Formal analysis; investigation; writing—review and editing. **Frederik Rask Dalby**: Conceptualization; methodology; supervision; writing—review and editing.

## CONFLICT OF INTEREST STATEMENT

The authors declare no conflicts of interest.

## Supporting information



Supplemental Figure S1. Physicochemical characterization of materials across production batches
Supplemental Figure S2. The effect of inoculum amount on CH_4_ emissions
Supplemental Figure S3. CH_4_ distribution and instrument calibration performance.
Supplemental Figure S4. Cumulative CH_4_ emissions from fresh and freeze/thawed inoculum
Supplementary Text S1. TN determination and chemical analyses
Supplementary Text S2. Parameters setting in UHPLC System and Mass spectrometry conditions
Supplemental Table S1. Classification criteria for metabolite functional groups based on elemental ratios.

## Data Availability

Data are available from the authors upon reasonable request.
