# Supplementary material for: Influence of substrate freshness on metabolite dynamics and methane emissions from pig slurry during laboratory incubation
Source: J Environ Qual. 2026 Aug 6;55(4):e70235. doi: 10.1002/jeq2.70235 (PMC13447882; doi:10.1002/jeq2.70235)
Supplement: Supplementary file 1 [file JEQ2-55-0-s001.docx]

Supplemental Material

Influence of substrate freshness on metabolite dynamics and methane emissions from pig slurry during laboratory incubation

Authors: ^1^Rui Wang, ^1^Michael Jørgen Hansen, ^1^Sasha Daniel Hafner, ^1^Frederik Rask Dalby*

Affiliations: Aarhus University, Department of Biological and Chemical Engineering, Denmark

Corresponding author*

Email: fd@bce.au.dk

The Supplemental Material contains:

Pages: 8

Tables: 1

Figures: 4

Text: 2

Physicochemical characterization of materials across production batches

Figure S1. Physicochemical characterization of feces, urine, and bulk slurry collected from the same farm across multiple production batches at the same growth stage. pH is dimensionless. TS is expressed as % of fresh weight. VS, NDF, cellulose, hemicellulose, lignin, and lipids are expressed as % of TS. TN, TAN, and VFA are expressed as g kg^-1^ slurry. Box plots show the median, interquartile range, and range of values across batches (n = 4).

Supplemental Figure S2. The effect of inoculum amount on CH_4_ emissions

Figure S2. Cumulative CH_4_ emission over time under different inoculum levels. FU0: feces, urine without inoculum; FU25: feces, urine with 25% inoculum; FU50: feces, urine with 50% inoculum.

Supplemental Figure S3. CH_4_ distribution and instrument calibration performance.

Fig.S3 Histogram showing the frequency distribution of CH_4_ concentrations (ppm) across all observations(A). Mass flow meter calibration (B). Picarro calibration using certified CH_4_ standard gases (C). Mass flow controller calibration verified through controlled dilution of certified CH_4_ standard gases (D).

Supplemental Figure S4. Cumulative CH_4_ emissions from fresh and freeze/thawed inoculum

Fig.S4 Cumulative CH_4_ emissions from fresh (yellow circle) and freeze/thawed (blue square) inoculum incubated alone at 20°C over 30 days. Data are presented as mean ± SD (n = 3). Fresh inoculum was used within 12 hours of collection, while frozen inoculum was stored at -18°C for three months prior to use.

Supplementary Text S1. TN determination and chemical analyses

TN was determined with two different methods depending on the sample composition. For treatments containing inoculum, TN was measured directly using Tveskaeg Benchtop low-field NMR sensor (NanoNord A/S, Aalborg, Denmark). TN was measured as TKN. TKN was measured using a Kjeltec 8400 system (FOSS, Denmark). For TAN and VFAs analysis, 1.000 ± 0.001 g of slurry was accurately weighed and diluted 50 – 70 times with deionized water. Total ammonia nitrogen (TAN) was determined using a photometric method with the Spectroquant Ammonium Test kit (Supelco, Merck, Darmstadt, Germany), and TAN was quantified photometrically with a Spectroquant NOVA 60 system (Merck, Germany). VFAs were quantified using a gas chromatograph (HP 6850 Series, Agilent Technologies, USA) equipped with a flame ionization detector and an HP-INNOWax column (30 m × 0.25 mm × 0.25 μm). For the CH_4_ measurements in the free acetic acid inhibition experiment, CH_4_ was quantified using a gas chromatograph equipped with a thermal conductivity detector (GC-TCD) and a ShinCarbon ST 80/100 packed column (2 m, 1/8" OD; Restek Corporation, United States), with argon employed as the carrier gas.

Supplementary Text S2. Parameters setting in UHPLC System and Mass spectrometry conditions

A Vanquish UHPLC System was coupled online to an Orbitrap Exploris 120 mass spectrometer (Thermo Fisher Scientific) for the analysis. Viper capillaries were used for all connections, including the sample injection valve, column inlet, and the probe of the OptaMax NG heated electrospray ionization source (H-ESI). The injection volume was 10 μL, and a 25 μL sample loop was employed in direct injection mode with the analytical-flow LC system. The chromatographic separation was performed using a BEH C18 Waters column with a guard column (1.7 μm particle size, 2.1 mm ID × 100 mm). The column temperature was maintained at 35 °C. Mobile phases A and B consisted of 0.1% formic acid in LC-MS grade water and 0.1% formic acid in acetonitrile, respectively. A gradient elusion was conducted at a flow rate of 0.250 μL/min, starting with 3% B and increasing to 95% B over 15 minutes, followed by 95% B for 5 minutes. A 3-minute equilibration period at initial conditions was applied before each injection.

The mass spectrometer was operated in positive polarity mode with a full scan mass range of 50 – 700 m/z. Key parameters for the Orbitrap Exploris 120 mass spectrometer were as follows: spray voltage, 3.5 kV; vaporizer temperature, 300 °C; ion transfer tube temperature, 320 °C; and funnel RF lens value, 70. Sheath gas, auxiliary gas, and sweep gas flow rates were set to 40, 8, and 1, respectively. Data acquisition employed data-dependent acquisition (DDA) with a Full MS-ddMS2 setup and a Top N value of 4 (number of dependent scans). The Full MS resolution was set to 60,000 at m/z 200, and the AGC target was set to standard. Dynamic exclusion was applied with an exclusion duration of 2.5 seconds, a mass tolerance of 5 ppm, and an intensity threshold of 5 × 10^3^. MS2 spectra were acquired at a resolution of 30,000 with a normalized collision energy of 30%, an isolation window of 1.5 m/z, and a maximum injection time of 22 ms. Both MS1 and MS2 spectra were recorded in profile mode.

Supplemental Table S1. Classification criteria for metabolite functional groups based on elemental ratios.

| Category | H/C Range | O/C Range | N/C Range | P/C Range | Additional Notes |
| --- | --- | --- | --- | --- | --- |
| Lipids | 1.32–2.00 | 0.00–0.60 | — | — | — |
| Lignin/CRAM-like | 0.70–1.50 | 0.10–0.67 | — | — | — |
| Carbohydrates | 1.50–2.40 | 0.67–1.20 | — | — | — |
| Unsaturated Hydrocarbons | 0.70–1.50 | 0.00–0.10 | — | — | — |
| Protein/Amino Acids/Peptides (refined) | See below | See below | ≥ 1 | < 0.17 | See refined criteria below |

Refined Criteria for Protein/Amino Acids/Peptides (applied after initial classification)

Condition 1: N ≥ 1, N/C = 0.1–0.3, O/C = 0.2–0.8, H/C = 1.2–2.2

Condition 2: OC = 0.12–0.6, HC = 0.9–2.5, NC = 0.126–0.7, PC < 0.17, N ≥ 1

OR OC = 0.6–1.0, HC = 1.2–2.5, NC = 0.2–0.7, PC < 0.17, N ≥ 1

Condition 3: OC = 0.3–0.5, HC = 1.5–2.0, NC = 0.2–0.4, N ≥ 2
